# Supplementary material for: Relationship Between Clinical Factors and the Risk of Cerebral Vasospasm Following Aneurysmal Subarachnoid Hemorrhage: An Exploratory Analysis
Source: Life (Basel). 2025 Dec 30;16(1):59. doi: 10.3390/life16010059 (PMC12843045; doi:10.3390/life16010059)
Supplement: Supplementary file 1 [file life-16-00059-s001.zip › life-4063204-supplementary.pdf]

Supplementary Table S1. Key statistical measures (including quartiles, interquartile range, and outlier information) for various clinical factors related to the risk of cerebral vasospasm, highlighting the distribution and variability of data among patient observations.

variable	***Q1***	***Q3***	***IQR***	***lower_bound***	***upper_bound***	***n_non_na***	***n_outliers***	***pct_outliers***
Age	46,75	62,25	15,50	23,50	85,50	44	0	0,00
CRP	4,35	48,55	44,20	-61,95	114,85	43	2	4,65
HTC	32,08	39,65	7,58	20,71	51,01	44	0	0,00
K	3,73	4,08	0,36	3,19	4,61	43	2	4,65
Na	138,00	143,25	5,25	130,13	151,13	44	1	2,27
WBC	9,21	13,22	4,02	3,19	19,25	44	2	4,55
WFNS grade	1,00	5,00	4,00	-5,00	11,00	44	0	0,00
baseline mRS	2,00	5,00	3,00	-2,50	9,50	44	0	0,00
creatinine	50,75	68,00	17,25	24,88	93,88	44	2	4,55
discharge mRS 90 day	2,00	5,00	3,00	-2,50	9,50	44	0	0,00
karbamid	2,86	5,60	2,74	-1,26	9,71	44	0	0,00
lymphocyte	1,06	2,03	0,97	-0,40	3,48	43	0	0,00
neutrofil	6,77	10,72	3,96	0,83	16,65	44	2	4,55
platelet	180,25	288,00	107,75	18,63	449,63	44	0	0,00
